# Multiple triangulation and collaborative research using qualitative methods to explore decision making in pre-hospital emergency care

**DOI:** 10.1186/s12874-017-0290-z

**Published:** 2017-01-24

**Authors:** Maxine Johnson, Rachel O’Hara, Enid Hirst, Andrew Weyman, Janette Turner, Suzanne Mason, Tom Quinn, Jane Shewan, A. Niroshan Siriwardena

**Affiliations:** 1 Public Health Section, ScHARR, University of Sheffield, Sheffield, UK; 2 Sheffield Emergency Care Forum, Sheffield, UK; 3 Department of Psychology, University of Bath, Bath, UK; 4 Emergency & Urgent Care Research, Health Services Research Section, ScHARR, University of Sheffield, Sheffield, UK; 5 Emergency Medicine, Health Services Research Section, ScHARR, University of Sheffield, Sheffield, UK; 6 Centre for Health and Social Care Research, Kingston and St George’s University, London, UK; 7 Yorkshire Ambulance Service NHS Trust, Wakefield, UK; 8 Primary and Prehospital Health Care, Community and Health Research Unit, College of Social Science, University of Lincoln, Brayford Pool, Lincoln, UK

**Keywords:** Multi-method research, Ethnography, Qualitative, Patient safety, Pre-hospital, Ambulance service

## Abstract

**Background:**

Paramedics make important and increasingly complex decisions at scene about patient care. Patient safety implications of influences on decision making in the pre-hospital setting were previously under-researched. Cutting edge perspectives advocate exploring the whole system rather than individual influences on patient safety. Ethnography (the study of people and cultures) has been acknowledged as a suitable method for identifying health care issues as they occur within the natural context. In this paper we compare multiple methods used in a multi-site, qualitative study that aimed to identify system influences on decision making.

**Methods:**

The study was conducted in three NHS Ambulance Trusts in England and involved researchers from each Trust working alongside academic researchers. Exploratory interviews with key informants e.g. managers (*n* = 16) and document review provided contextual information. Between October 2012 and July 2013 researchers observed 34 paramedic shifts and ten paramedics provided additional accounts via audio-recorded ‘digital diaries’ (155 events). Three staff focus groups (total *n* = 21) and three service user focus groups (total *n* = 23) explored a range of experiences and perceptions. Data collection and analysis was carried out by academic and ambulance service researchers as well as service users. Workshops were held at each site to elicit feedback on the findings and facilitate prioritisation of issues identified.

**Results:**

The use of a multi-method qualitative approach allowed cross-validation of important issues for ambulance service staff and service users. A key factor in successful implementation of the study was establishing good working relationships with academic and ambulance service teams. Enrolling at least one research lead at each site facilitated the recruitment process as well as study progress. Active involvement with the study allowed ambulance service researchers and service users to gain a better understanding of the research process. Feedback workshops allowed stakeholders to discuss and prioritise findings as well as identify new research areas.

**Conclusion:**

Combining multiple qualitative methods with a collaborative research approach can facilitate exploration of system influences on patient safety in under-researched settings. The paper highlights empirical issues, strengths and limitations for this approach. Feedback workshops were effective for verifying findings and prioritising areas for future intervention and research.

## Background

Delivery of pre-hospital emergency care takes place in a complex environment where front-line ambulance service (AS) staff are now faced with decision-making over an array of patient care options. For example, rather than simply making a decision to convey patients to an Emergency Department (ED), paramedics can treat patients at scene, refer patients to another service or convey them to a non-emergency service care provider. In the UK, the National Health Service (NHS) comprises a number of ‘Trusts’ that manage hospital, primary, mental health and ambulance service care. There are ten Ambulance Trusts in England, whilst ambulance services in Wales, Scotland and Northern Ireland are each covered by one Trust. Decisions made by paramedics during the course of responding to emergency calls have the potential to impact on patient health outcomes as well as embodying professional risk for individual staff members [1] and reputational risk to the employing Trust. These decisions are made in the context of organisational constraints, changes in patient demographics and evolving professional roles for paramedics [2]. Advanced roles and specialist training have developed in an attempt to manage increasing demand from a broad range of patients with non-life threatening conditions where healthcare needs may be more suited to community and/or social care rather than the Emergency Department (ED) [3]. To fully understand the nature of paramedic decision making in this context and potential threats to patient health outcomes, our research examined macro, meso and micro level systemic influences, in other words, those that are at the organisation, community and person levels [4]. This paper details and reflects on this research conducted within the complex environment of pre-hospital emergency care. The aim of the paper was to describe the methodological approach employed in this study in order to share lessons on collaboration in multi-method research across multiple sites and investigators.

In line with established approaches to patient safety [5], we aimed to explore systemic influences on decision making that represent potential threats to patient safety rather than assessing the apparent safety of individual practitioner decisions. We were particularly interested in decisions made in relation to transition points in the patient care pathway, i.e. where a patient is discharged with advice, referred to another service or conveyed to hospital. Details of the study and findings are reported elsewhere [6].

There are few published multisite studies using multiple qualitative methods and organisations to examine the dynamic and mobile pre-hospital emergency context of ambulance services. Previously published papers provide only brief information about methods used to carry out the research and the lessons learned. This study was conducted in three Ambulance Service Trusts in England, representing a variety of contextual factors in the prehospital emergency care system (e.g. care pathways, staff roles, service configuration). The use of multiple qualitative methods allowed the findings from each method to bring particular insights and understanding, including real-time observation of events and reconstructed accounts of events during interviews and focus groups [7].

The geographical area covered by each Trust included densely populated urban areas, sparsely populated rural areas, coastline and busy stretches of motorway. The study comprised three phases. Phase 1 aimed to develop a contextual understanding of each site. Phase 2 examined decision making by paramedics across the three Ambulance Trusts using an ethnographic approach to study their actions and accounts in everyday context [8]. The ethnographic approach uses a range of methods which usually includes observation, to study people and cultures. Ethnography has previously been utilised in healthcare research for assessing collaborative practices, [9] management cultures [10] and decision making practices in relation to mental health [11]. Phase 3 involved feedback workshops at each site.

The multi-disciplinary research team for the study reported here encompassed expertise in social science, emergency care research, risk and patient safety and included representation from healthcare professionals in the Ambulance Service, Emergency Department and primary care, as well as service users. Our aim was to identify systemic influences on paramedic decision making with regard to their potential impact on patient safety. In this paper we describe salient aspects of the research process as well as critically assessing the utility of our approach for addressing the research question.

## Methods

**Fig. 1:**
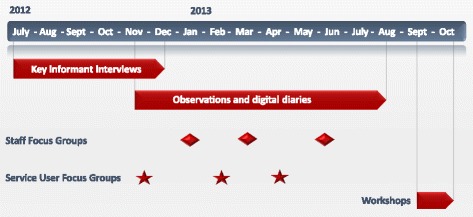
Data collection timeline

A primarily qualitative approach was chosen as being best suited to the exploratory nature of the study and to address the research question. In order to maximise credibility, dependability and confirmability of the findings [12]. We adopted a multiple site, multiple method and multiple investigator design across three phases of the research. The qualitative methods employed in this study included document review, interviews, observation, digital diaries, focus groups and workshops to afford a more thorough and multi-faceted examination of issues than could be gained from any single method. Figure 1 provides a timeline of the multiple methods used to collect data.

The study, conducted in three ambulance service Trusts in England, involved the recruitment of clinical and non-clinical based researchers to the study from each Trust to work alongside the academic researchers. Staff and service user participants at each site were recruited through available Ambulance Service and Patient and Public Involvement networks as described for each phase of the study.

### Data collection

#### Phase 1: Understanding the context

The aim of the initial phase of the study was to gain an understanding of the organisational context within each Trust and elicit perceived influences on transition decisions and associated patient safety issues. This involved conducting semi-structured interviews with a small number of key ambulance service staff (*n* = 16) who could provide an overview of the organisation and service delivery, including clinical governance leads, directors of operations, medical or clinical leads as well as paramedics with dual operational and managerial or leadership roles. We also collected demographic site information and relevant documentation on policies and procedures. This allowed us to provide context for each Trust in terms of area, population and operational work volumes as well as understanding relevant national and local policies.

#### Phase 2a: Paramedic decision making in practice

The main phase of the study entailed non–participant field observation of paramedics’ day to day working practices in order to gain insight into potential systemic impacts on conveyance decisions, for example, whether to transport to hospital. At each site Ambulance Service staff were recruited to the study as researchers to assist in recruitment, data collection and analysis. These were either operational paramedics (*n* = 2) or Trust research staff (*n* = 2). In one Trust, two members of ambulance service staff shared the research role. Thus, a total of four ambulance service staff carried out the research as well as one member of academic staff. A key function of involving ambulance service staff as researchers was to complement the academic researcher data to capture possible ‘insider’ and ‘outsider’ perspectives [8] on paramedic activity and systemic influences. Employing observers from a range of backgrounds also dilutes tendencies toward ‘going native’ [8] or conversely, being so overwhelmed by new information that subsequent data is meaningless. The academic researcher had a background in nursing which meant that she was familiar with the medical context though not pre-hospital emergency care.

In order to enhance the robustness of the approach, a familiarisation and pilot observation shift was carried out initially by two of the researchers (one ambulance service and one academic researcher). Subsequent refinement facilitated the development of a data collection template to give focus to the observations and information recorded. Details recorded included date, time, geographical setting and crew roles as well as ‘observational dimensions’ [13], such as activities at each patient attendance and any other people present, for example, professional emergency services, carers and bystanders.

Following each patient attendance, mini-interviews of variable duration with paramedics were carried out to establish the reasoning behind the decisions made [13]. These were recorded in written field notes or using an audio-recorder, the choice of method being a product of prevailing practical limitations. For example, it was often challenging to audio-record short sections of conversation that could be interrupted at any time due to work requirements, therefore this method of recording tended to be used for mini-interviews that were carried out during quieter moments.

A total of 36 paramedics (see Table 1) were observed between October 2012 and June 2013 over 34 shifts of 8–12 h duration. Observations were conducted over a 9 month period, on different days of the week and times of the day, including night shifts in order to gain insight into variability in work rates, types of care/treatment, types of patient.

**Table 1:** Staff roles of participants across sites

Staff Role	Site 1			Site 2			Site 3		
	*Obs*	*DD*	*FG*	*Obs*	*DD*	*FG*	*Obs*	*DD*	*FG*
Paramedic	13	3	6	13	2	7	7	2	4
Specialist paramedic (ECP, CCP, PP, CP)	2	-	2	1	1	1	3	2	1
Emergency care assistant/technician/support worker	6	-	-	6	-	-	6	-	-

*Obs* observations, *DD* digital diaries, *FG* focus groups, *ECP* emergency care practitioner, *CCP* critical care paramedic, *PP* paramedic practitioner, *CP* community paramedic

In order to supplement data from the observation shifts and to explore decision making events that might not have been observed during the shifts, ‘digital diaries’ (audio-recorders) were used to collect additional data from 10 paramedics (see Table 1). Each diary consisted of recordings describing a number of care decisions that were perceived as being challenging, and/or highlighting a potential threat to patient safety. The value of this method of data collection is that it allows participants to voice their experiences and concerns privately, immediately following an event and without intervention from researchers. The digital audio-recorders were distributed and collected by researchers at each site. A total of 13 paramedics initially consented to participate using the digital diary method. Three subsequently dropped out due to ill-health (*n* = 1) and feeling self-conscious about speaking into the audio-recorder (*n* = 2).

#### Phase 2b: Paramedic focus groups

We chose focus groups as a complementary method to observations and interviews. Focus groups allow researchers to identify sub-cultures within groups of staff with different roles [14] which would not necessarily have been evident during observations, given that much paramedic work is carried out as a sole worker or in pairs. Focus groups also added to the interview method of data collection in their generation of dialogue around experiences and perceptions [15]. Focus groups encourage participants to explore each other’s views, which can lead to a more detailed exploration of ideas than in one-to-one interviews [14].

We invited paramedics to participate in one focus group at each site in order to explore shared experiences, perspectives and decision criteria. The range of attendance at each focus group was between five and eight participants with a total of 21 staff attending groups across the three Trusts. The use of a topic guide encouraged a broader discussion of issues that had been raised during the Phase 1 interviews [14]. The focus groups also included a discussion of different aspects of ambulance personnel patient safety culture in their respective organisations using the Manchester Patient Safety Framework (MaPSaF) as a guide [16]. The MaPSaF tool is designed to help healthcare organisations and teams assess their progress in developing a positive safety culture. It uses critical dimensions of patient safety that relate to areas where attitudes, values and behaviours about patient safety are likely to be reflected in the organisation’s working practices, for example, how patient safety incidents are investigated, staff education, and training in risk management [17]. The tool provided a framework for discussion following the initial less structured discussion of Phase 1 findings. Engaging with the MaPSaF tool enabled participants to reflect on and discuss perceptions in pairs before exchanging views within the wider group. Focus groups were facilitated by the academic researchers with an ambulance service researcher present. Discussions were audio-recorded.

#### Phase 2c: Service user focus groups

Focus groups were also conducted with service users recruited through established Patient and Public Involvement (PPI) networks at two of the sites and more widely from community networks at the third site. Contacts within each service user representatives group assisted in the recruitment of participants. Between 7 and 8 service users attended each focus group, giving a total of 23 participants (see Table 2). Attendees across the sites comprised 11 females and 12 males across all adult age groups, although 16 (69.5%) were aged over 55 years. They represented a range of perspectives and experiences, including local PPI networks, long-term conditions, mobility issues and communication issues.

**Table 2:** Characteristics of service user focus group participants

Number of service user participants	Site 1 *N* = 7	Site 2 *N* = 8	Site 3 *N* = 8
Age range:			
18–24 years	1	0	0
25–34 years	0	1	0
35–44 years	0	2	0
45–54 years	1	1	1
55–64 years	3	2	4
65+ years	2	3	3
Male	3	5	4
Female	4	3	4

#### Phase 3: Feedback and prioritisation workshops

The final phase of the study entailed delivering stakeholder feedback workshops at each site. Participants from all phases of the study were invited to attend. Additional staff and service user representatives were also invited by each of the Ambulance Service Trusts. A total of 45 ambulance service staff and service users attended the three workshops. In addition to providing feedback on the study findings, the workshops provided an opportunity for further articulation of topics arising from the field work findings. Participants also performed a ranking exercise designed to capture perspectives for future risk reduction and enhanced service delivery intervention. The ranking took the form of individual judgements of the systemic issues identified in Phase 2 using the method of paired comparisons. To simplify what would otherwise be a cognitively challenging task, the method of paired comparisons requires each respondent to compare each priority issue, for example, ‘resources’, was paired with each of the other six issues (‘access to care’, ‘training and development’, ‘communication and feedback’, ‘demand’, ‘risk aversion’ and ‘performance regime’), one pairing at a time, for all permutations of pairings; in each case being asked to indicate which of each pair is deemed the more important [18].

### Data analysis

All audio-recordings, field notes and researcher diaries were fully transcribed. This generated a significant amount of data across researchers, sites and methods. As the data collection was conducted in sequential blocks of time at each site, the initial data analysis followed a thematic approach. Thematic analysis was chosen as it allowed data collected using a range of different qualitative methods to be analysed in a similar way. Data from each case (interview, focus group or observation session) was initially coded to provide a number of categories which were then grouped thematically across cases and compared to ensure that themes incorporated all relevant data. Thematic analysis can be carried out at a superficial level or it can be further developed to include explanatory interpretations and can also be used to develop taxonomies [19]. Subsequent analysis of the findings across the three sites revealed a high degree of consistency and commonality. In recognition of the high degree of homogeneity it was considered appropriate to pool the data.

Analysis of the Phase 2 data was managed using Atlas ti. ^TM^ qualitative data analysis software [20]. This supported the generation of a taxonomy of transition decisions and systems and structural influences with potential to impact on ambulance crew decision making with implications for patient safety. We began analysing the observation and digital diary data by transferring data from transcriptions into site and shift specific charts that represented each attendance together with the corresponding decision and ambulance crew account of their rationale for the decision. Patterns detected within the data revealed a typology of nine decisions encountered by paramedics representing a continuum of increasing complexity [6, 21]. Repeated iterations of coding identified a range of cues that were utilised during decision making as well as potential mitigating and mediating system and structural influences on these decisions. An equivalent approach was adopted for analysis of the paramedic focus group data which was analysed thematically. Analysis of service user focus group data identified areas of concern in relation to potential influences on ambulance service care and patient safety.

All Phase 2 staff data were analysed initially using an inductive approach [21] which begins close to the data and moves through levels of more abstract analysis to identify patterns and relationships that can be used to explain phenomena. This approach is in contrast to deductive analysis which begins with a supposition that is tested through analysing the data. In our analysis, similarities and differences across the various data collection methods were also explored. Although some differences and contradictions were identified these generally represented local area variations within individual sites rather than between sites. The global picture that emerged was notable consistency across the three sites and between methods. Findings from this inductive analysis were then mapped against Vincent’s Human Factors framework [22], which resulted in over 20 system influences across the various system levels. We found that the hierarchy of influences on clinical practice within this framework was not ideally suited to extracting more overarching themes from our data, which encompassed different levels of the hierarchy. To take account of this, we continued to analyse the data through repeated iterations until seven overarching issues emerged. The set of issues encompassed overlapping rather than distinct macro, meso and micro level influences [6]. At regular intervals the emerging themes were shared and discussed with the wider research team to ensure verification of findings and their interpretation.

**Fig. 2:**
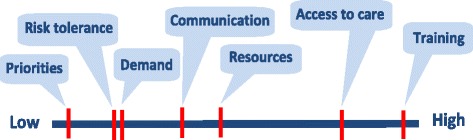
Relative importance of system issues represented on a single continuum

The analysis of the data from the workshop intervention prioritisation exercise was quantitative, following established practice for the method of paired comparisons. This involved checks of within respondent consistency (Kendall’s K) to determine whether participants could reliably form consistent judgement between the items; and the degree of concordance between respondents (Kendall’s W) [23]. This analysis showed a high level of internal reliability with 84% of respondents (*N* = 44) demonstrating a coefficient (K) of > 0.70. A high level of concordance between ambulance service staff and service user participants (Rho = 0.91) meant that it was acceptable to combine the data for each group [23]. Transformation of the rankings for the seven issues to standard values (z scores) produced the interval scale along which are plotted the issues presented to participants in respect of their prioritisation. Thus, the scale shows that staff perceived training and development as requiring the highest priority (see Fig. 2).

### Ethics

The project received ethical approval for the research involving NHS Ambulance Trust staff from the University of Sheffield Research Ethics Committee (ScHARR 0530/CAO) on the basis that no patient identifying information would be collected during the observations as we were examining paramedic decisions but not patient views at that time. The NRES Committee Yorkshire and the Humber—South Yorkshire (12/YH/0327) provided approval for the research involving service users. All participants were provided with information about the study prior to the consenting process.

Other ethical issues concerned data protection and security which were addressed by maintaining the University code of conduct in respect of storing data only within specified permitted access drives and using encrypted hardware. The safety of observers was optimised by adhering to the codes of practice at each site which, for example, prescribed high visual and safety attire during operational shifts as well as the University policy for lone researchers. The latter was particularly important for this study where researchers were travelling outside of normal office hours to unfamiliar locations. Where appropriate during the observational activity, researchers were identified to patients, carers and other health professionals encountered during observation shifts as observing the paramedics.

Observers needed to ensure that paramedics could practice without any interruption or distraction that could affect patient care. Similarly, we acknowledged that operational demands were paramount and should take precedence throughout the study. For example, on two occasions interviews were cancelled to take account of high seasonal operational demands. Given the relatively small sample at each site and the fact that participating paramedics were known to colleagues and managers; when reporting the findings, care was taken to protect the identities of individuals. Phase 1 interviewees were informed as part of the consenting process that due to the small number of key staff being interviewed at each site, confidentiality or anonymity could not be guaranteed.

## Results

The study highlighted two key lessons regarding multi-method, multi-disciplinary collaborative research. Although we found that the use of multiple methods, sources and investigators to obtain data across sites was insightful it added to the complexity of the design, and embodied time penalties. This is considered to have been more than offset by the benefits arising from continual collaboration between academic researchers, the ambulance service Trusts and service user representatives and was a valuable feature of the research process. We now reflect upon these lessons.

1. *Multi*-*triangulation*: *the contribution of multiple methods*, *sources and investigators*

**Fig. 3:**
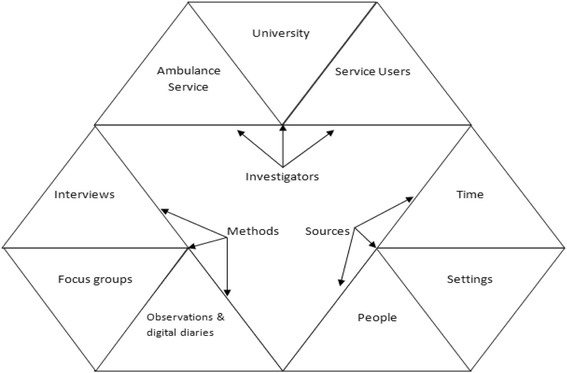
Applying multi-triangulation to understanding systemic influences on paramedic decision making

Our approach adopted a multiple triangulation approach as described by Denzin [24] to incorporate multiple methods of data collection, multiple sources of data and multiple investigators with multiple areas of expertise. We present these methods, sources and investigators and the relationship between them in Fig. 3. The multi-site and multi-method design facilitated the identification and validation of relevant issues. Denzin [24] states that multiple research methods are desirable because each method reveals a different aspect of reality. This idea has since been developed to include triangulation as a metaphor for strength [25], trustworthiness [12], and comprehensiveness [26]. Guba [12] argues that trustworthiness through triangulation enhances the credibility, dependability and ‘confirmability’ in qualitative studies.

### Method triangulation

We used a range of qualitative research methods to collect data from each participating site, including document review, interviews, observations, digital diaries and focus groups. Each research method was chosen to access different types of information for comparing findings across methods. For example, an issue that was identified during an interview or focus group could also be examined during observations of practice in the naturalistic work setting. Similarly, issues witnessed during observations or recorded in digital diaries could be explored during discussions. The workshops with Ambulance Service staff and service users provided a further level of verification. The data collection methods that were used and their respective advantages and limitations are presented in Table 3.

**Table 3:** Multiple methods used and their contribution to the project

	Advantages	Limitations
Interviews	Relatively easy to organise and carry out.	Reliant on ability of participant to recall information.
	Researcher can probe to clarify meanings or obtain depth of information.	Provides only one perspective so sample needs to incorporate diversity.
Observations	Produces real time data, not reliant on accurate recall.	Time consuming; researcher needs to spend long periods of time in the setting.
	Researchers can validate or question information obtained from other methods.	Much of data repetitive; can only report events happening during the observation period.
Digital Diaries	Allows participants to provide recent data without interaction of researcher.	Some participant reluctance to speak into an audio-recorder.
	Participants can choose when to participate.	Researchers unable to probe to find meaning in data.
Focus Groups	Encourages interaction between participants which can stimulate further discussion.	Reliant on ability of participant to recall information and have the confidence to contribute to a discussion.
	Facilitates discussion of shared experiences such as workplace culture or service user issues.	Requires sufficient numbers of participants available at the same time.
Stakeholder workshops	Allows participants and other stakeholders to discuss and provide feedback on findings.	Requires large, accessible venue and adequate facilities.
	Allows group prioritisation of implications for research and intervention.	Requires sufficient numbers of participants available at the same time.

### Source triangulation

Obtaining information from a range of sources, across settings and time was an important aspect of the data collection activity. This supported our objective to analyse the data for site specific, role specific and time specific features that might affect decision making. This included identifying practices that varied according to different Ambulance Service Trust protocols or local initiatives. Variation in paramedic responsibilities and roles can occur where those with advanced training undertake more specialist activities. Potential temporal influences include fluctuations in demand for emergency care and access to referral pathways. Observations were conducted across the variety of different paramedic shifts (times and days) to accommodate any potential variation in patient demographics, decision support and decision options.

### Investigator triangulation

We included a range of investigator perspectives so that the findings were not based solely on academic researcher interpretations of events and themes. The involvement of ambulance service researchers in data collection and analysis allowed verification of findings from a range of perspectives. Similarly, a service user member of the research team contributed to the thematic analysis of the data from the service user focus groups. Including participation in data collection and interpretation from a range of investigators ensured that a variety of perspectives were taken into account. Involvement of the wider project team which encompassed a range of disciplines and perspectives is described in more detail later in the findings.

A key reported benefit from multiple triangulation is increased confidence in the findings. [27] The field observations highlighted the potential complexity of the emergency decision making context. Changes in the profile of public demand for ambulance services and structural changes in health care provision have combined to increase the range of care options that paramedics need to consider and in instances where the need for conveyance to an emergency department (ED) is not obvious, they are expected to consider alternative care pathways. These changes have had the effect of increasing the complexity of paramedic decision making, and also brought logistical challenges, for example, due to geographical variability in non-emergency care provision, or encountering resistance to accept patients by some care providers [5].

### Integration of data from multiple methods, sources and investigators

Documents from Phase 1 were reviewed to increase our understanding of the organisational context for the study. We organised Phase 1 interview data using Atlas. ti 7™ [20] software and to conduct a thematic analysis. Themes from this phase were used as the basis for the discussion guides used during paramedic and service user focus groups. Data from Phase 2 (observations, interviews and digital diaries) was iteratively coded and categorised. Focus group data were then analysed using the constant comparison method [6, 21]. Apparent variations in data collected by different researchers were also examined. However it was challenging to make inferences in this respect as the data differed in other ways such as style of reporting and extent of detail included. Working to a broad observation template [13] helped to maintain a minimal degree of consistency in scope across observations and observers.

2. *Maintaining collaborative working relationships to achieve study objectives*.

Developing good working relationships between academic researchers and ambulance service research staff at each site was crucial to achieving the study objectives. During the design and implementation stages of the study we discussed practical and theoretical issues with ambulance service research managers, university academics and patient and public involvement (PPI) representatives. This allowed a broad range of perspectives to be accounted for in the study protocol as well as increasing our understanding of contextual elements and any service user considerations prior to enquiry. Initial meetings and discussions began a phase of ‘prolonged engagement’ within the setting during which researchers could become more familiar with the previously unfamiliar [12]. This would pave the way for data collection and help to maintain good working relationships throughout the study. The study team included researchers and clinicians in emergency care, from hospital and pre-hospital settings. Ambulance service collaborators were also vital to understanding pertinent contextual issues and negotiating issues relevant to ethics and local governance approvals.

Credibility of the findings was essential [12], so we regularly discussed interim data and emerging themes within the wider team to clarify our understanding. The study was dependent upon key people from within the three Trusts to facilitate access to study participants. The ambulance service research managers at each site assisted in obtaining relevant organisational documentation and accessing potential interview participants for Phase 1. They also assisted in the recruitment of ambulance service researchers to the study team for Phase 2 activities.

In order to ensure that the study findings would be transferable [12] rather than context dependent, the study was designed to include the perspectives and experiences of a broad range of ambulance service and service user informants. Paramedics taking part in Phase 2 were recruited from at least two different geographical areas within each Ambulance Service Trust to maximise potential variation in service demands and delivery.

A range of complementary methods were used to communicate details of the study to potential participants, including invitations and information sheets sent via email, and flyers via organisational intranet. Having an ambulance service researcher within each site was important in facilitating study progression during Phase 2 and overcoming one of the major challenges for recruitment and data collection—communicating with paramedics who were ‘out on the road’. Having office based researchers with knowledge of the operational environment and personnel within their organisation was key to the data collection process. They were able to liaise with paramedics and managers in ambulance stations across a number of different ambulance service areas. They were also instrumental in arranging suitable venues for the focus groups and workshops. In one of the sites, liaison with participants to arrange observations was facilitated by the member of staff that arranges placements for trainees.

### Collaborating with ambulance service staff

The paramedic role has undergone significant change over the past decade, including professional registration, specialist practice and a growing number of paramedics undertaking higher education courses and becoming involved in research. A benefit of our research collaboration for ambulance service researchers and participants was that they gained a more detailed understanding of the research process through participating in different aspects of the study. Potential challenges for ambulance service researchers included having a previous working relationship with the crews in their paramedic role within the same organisation. There was a concern that crews would behave differently when they were being observed by familiar members of staff. To some extent this was prevented by ambulance service researchers observing crews that worked in a different location to themselves. There was also a concern that researchers with a paramedic background might incur observer bias because they were so familiar with the context. However, the value of their understanding of the nuances of pre-hospital emergency care provided complementary insights to those of the other researchers. For example the explanation for a decision about which hospital to transport a patient to, might not be taken at face value by operational ambulance service researchers because of their prior knowledge about condition-specific pathways. A disadvantage of prior knowledge is that questioning may not occur where the observer makes assumptions about what is being observed or perceives that they know what the response might be. Non-clinical researchers faced the challenge of developing an understanding of the paramedic role, but their questioning should be more in depth because they are less prone to making assumptions.

Ambulance service researchers attended the staff and service user focus groups. Whilst they did not participate in discussions, their expertise was crucial in clarifying some of the issues being addressed, particularly for the service users. Similarly, during feedback workshops, ambulance service researchers were available to comment about their experiences during data collection. A key output of the workshops was that collaborative relationships developed between the academic and ambulance service teams over the duration of the study supported the identification of new ideas for future research. A range of potential avenues for further research were identified. Workshop attendees were asked to rank the seven headline issues that had been identified from our study as topics for future attention as well as identifying areas for future intervention and/or research. In seeking to prioritise issues we were mindful of the difficulty respondents can experience when they attempt to rate multi-faceted constructs and the propensity of rating scales to produce ceiling effects, where the items are all considered ‘important’. The method paired comparisons has the advantages of low cognitive load, formal testing of reproducibility and agreement between respondents, while also translating into an interval scale [23]. The two highest ranked priorities were training and development, and access to care. Both of these featured strongly in the recommendations for further research. Ongoing collaboration between members of the research team has enabled a number of research recommendations to be explored as part of a mutually agreed research agenda.

### Collaborating with service users

PPI was also central to the successful implementation of the study objectives. A local PPI panel focused on supporting emergency care research provided input at key stages from design to completion (e.g. reviewing ethics documentation). A member of this panel (EH) was engaged as a study co-applicant from the outset and actively contributed to the study design, service user recruitment, data collection, data analysis and dissemination stages of the research. Study outputs have included producing a lay leaflet to communicate the findings to the public, which have been distributed via PPI networks and local primary care services.

## Discussion

This study used multiple triangulation of methods, sources and investigators across three sites to explore macro, meso and micro systemic influences on pre-hospital decision making with potential impacts on patient safety. A collaborative approach was adopted between academic and ambulance service teams to develop and implement the study aims and objectives. An important factor in achieving the objectives in this multi-site study was the development of a relationship involving continual collaboration across the distributed network of research team members. This was further supported by enrolling a research lead at each site and using multi-triangulation and feedback throughout to generate and validate the research findings as well as identifying priority areas for service delivery improvement and further research.

Cooper et al. [28] provide a case for using ethnographic methods in the pre-hospital setting, stating that the approach allows the study of organisation or group culture. This study included observation of paramedics supplemented by mini-interviews where possible around responding to emergency calls to explore system constraints on decision making, including cultural influences. As Cooper et al. [28] point out, strict adherence to a particular theory can be unhelpful during analysis. Vincent’s Human Factors framework [22] was initially useful in this study for considering different aspects of the system that may influence paramedic decision making. However, the findings from this study did not fit neatly into the hierarchy of influences on clinical practice for this framework, with some issues on the interface between the different levels of the framework. A further iteration of inductive analysis identified overarching factors relevant to potential influences on decision making and patient safety.

Dixon Woods [29] also highlighted the ability of ethnography, to access information that might be unknown to those being observed, particularly in the area of patient safety. Combining methods in this study allowed researchers to speak to operational staff as well as observe crews whilst carrying out their decision making practices. It was found that during observation shifts practices were not observed to affect patient safety, but there was a potential for system impacts to influence decisions that might not be optimal for patients. In particular the varying degree of access to alternative options to ED across sites was notable.

A key strength of the study was the use of multiple methods to collect data, resulting in different ways of seeing reality [24], yet similar issues were highlighted in data generated by each method. The collaborative nature of the study strengthened the dissemination of findings from the study as well as opening avenues for collaboration on future research addressing ambulance service priorities.

Limitations of the study include the time taken to negotiate access to participating Trusts. Methods of obtaining access differed at each site, and a more standardised procedure might have improved efficiency in the early stages. It was also challenging to access operational staff ‘on the road’ to recruit to the study. Digital diary entries had to be interpreted without recourse to probing for further information. An additional limitation is the lack of availability of ‘outsider’ observer data at one site due to unforeseen circumstances. However given the challenges, working relationships with the three sites enabled most of the study aims to be met in a timely manner.

The study provided an overview of paramedic decision making, which is considered to be an under researched area, from which a number of issues were raised for further research and future service delivery [6]. Whilst anecdotally the issues raised may not be surprising to ambulance service staff, publishing this work was an important step in making these issues transparent for policy makers.

## Conclusions

Combining multiple qualitative methods with a collaborative research approach can facilitate exploration of system influences on patient safety in under-researched settings. Triangulation of data collection across time, from a range of roles in different settings, using a combination of research methods, carried out by investigators representing clinical, research and service user perspectives allowed different realities to be explored within the same study and enhanced the credibility of findings. A strong collaboration between academics and ambulance services and PPI representatives was crucial to achieving study aims. Feedback workshops were effective in supporting the verification of findings, as well as providing an opportunity to identify priority areas for intervention and research.

## Abbreviations

AS: Ambulance service
ED: Emergency Department
MaPSaF: Manchester Patient Safety Framework
NHS: National Health Service
NRES: National Research Ethics Service
PPI: Patient and public involvement
